# *m*-Xylylenediaminium sulfate: crystal structure and Hirshfeld surface analysis

**DOI:** 10.1107/S2056989016006940

**Published:** 2016-05-04

**Authors:** Afef Guesmi, Sofian Gatfaoui, Thierry Roisnel, Houda Marouani

**Affiliations:** a Laboratoire de Chimie des Matériaux, Faculté des Sciences de Bizerte, 7021 Zarzouna Bizerte, Université de Carthage, Tunisia; b Centre de Diffractométrie X, UMR 6226 CNRS, Unité Sciences Chimiques de Rennes, Université de Rennes I, 263 Avenue du, Général Leclerc, 35042 Rennes, France

**Keywords:** crystal structure, *m*-xylylenediaminium, sulfate, hydrogen bonding, Hirshfeld surface analysis, fingerprint maps

## Abstract

The crystal structure of the title salt {systematic name: [1,3-phenyl­enebis(methyl­ene)]bis­(aza­nium) sulfate}, C_8_H_14_N_2_^2+^·SO_4_^2−^, consists of infinite (100) sheets of alternating organic and inorganic entities The *m*-xylylenediaminium cations are linked to the sulfate anions by N—H⋯O and asymmetric bifurcated N—H⋯(O,O) hydrogen bonds, generating a three-dimensional network. A weak C—H⋯O inter­action also occurs. The Hirshfeld surface analysis and the two-dimensional fingerprint maps indicate that the packing is dominated by H⋯O/O⋯H and H⋯H contacts.

## Abstract 2

The crystal structure of the title salt consists of infinite (100) sheets of alternating organic and inorganic entities The *m*-xylylenediaminium cations are linked to the sulfate anions by N—H⋯O and asymmetric bifurcated N—H⋯(O,O) hydrogen bonds, generating a three-dimensional network. The Hirshfeld surface analysis and the two-dimensional fingerprint maps indicate that the packing is dominated by H⋯O/O⋯H and H⋯H contacts.

## Chemical context   

*m*-Xylylenediaminum compounds have been intensively investigated due to their good anti­microbial activity against various anti­bacterial and anti­fungal species (Murugesan *et al.*, 2015 ▸). Sequestration of carbon dioxide by *m*-xylylene­di­amine with formation of a crystalline adduct has been reported (Lee *et al.*, 2013 ▸). In addition, polyamides of *m*-xylylenedi­amine possess dielectric properties (Murata *et al.*, 1999 ▸). In this work, as part of our studies in this area, we report the synthesis, the structural investigation and the Hirshfeld surface analysis of a new organic sulfate salt, (C_8_H_14_N_2_)SO_4_, (I).
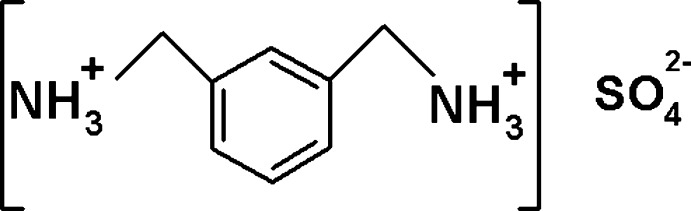


## Structural commentary   

**Figure 1:**
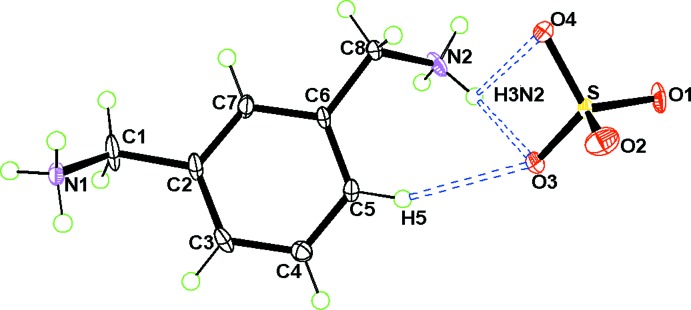
A view of (I), with displacement ellipsoids drawn at the 30% probability level. H atoms are represented as small spheres of arbitrary radii. Hydrogen bonds are shown as dotted lines.

The asymmetric unit of (I) comprises one *m*-xylylene­diaminium cation and one sulfate anion (Fig. 1 ▸). Both ammonium groups in the *m*-xylylenediaminium cation adopt a *trans* conformation with respect to the benzene ring. The same conformation has been observed in C_8_H_14_N_2_^2+^·2Cl^−^ (Cheng & Li, 2008 ▸), but in C_8_H_14_N_2_^2+^·2NO_3_^−^ (Gatfaoui *et al.*, 2014 ▸) the *cis* conformation occurs. Thus, the cation conformation is modified when substituting sulfate or chloride anions by nitrates. Examination of the organic cations shows that the bond distances and angles show no significant differences from those in other compounds involving the same organic groups (Cheng & Li, 2008 ▸; Gatfaoui *et al.*, 2014 ▸). The aromatic ring of the cation is essentially planar with an r.m.s. deviation of 0.0014 Å.

In the sulfate anion, the S—O bond lengths range from 1.4673 (12) to 1.4895 (11) Å. Their similar values confirm the absence of a proton in this anion. It is worth noting that the S—O4 distance is the longest because O4 accepts three hydrogen bonds, one of which is considered to be strong (Blessing, 1986 ▸; Brown, 1976 ▸). The average values of the S—O distances and O—S—O angles are 1.4799 Å and 109.46°, respectively. Similar geometrical features have also been observed in other crystal structures (Marouani *et al.*, 2011*a* ▸,*b* ▸). The calculated average values of the distortion indices (Baur, 1974 ▸) corresponding to the different angles and distances in the SO_4_ tetra­hedron [DI(SO) = 0.006, DI(OSO) = 0.008, and DI(OO) = 0.003] show a slight distortion of the OSO angles if compared to the SO and OO distances. Hence, the SO_4_ group can be considered as a rigid regular arrangement of oxygen atoms, with the sulfur atom slightly displaced from the centre of gravity.

## Supra­molecular features   

The packing of the title salt is dominated by hydrogen bonding, as detailed in Table 1 ▸. Ten distinct hydrogen bonds of types N—H⋯O and C—H⋯O involve all of the oxygen atoms of the sulfate anions as acceptors, However, only two of the N—H⋯O hydrogen bonds are considered as strong according to the Blessing and Brown criteria (Blessing, 1986 ▸; Brown, 1976 ▸).

**Figure 2:**
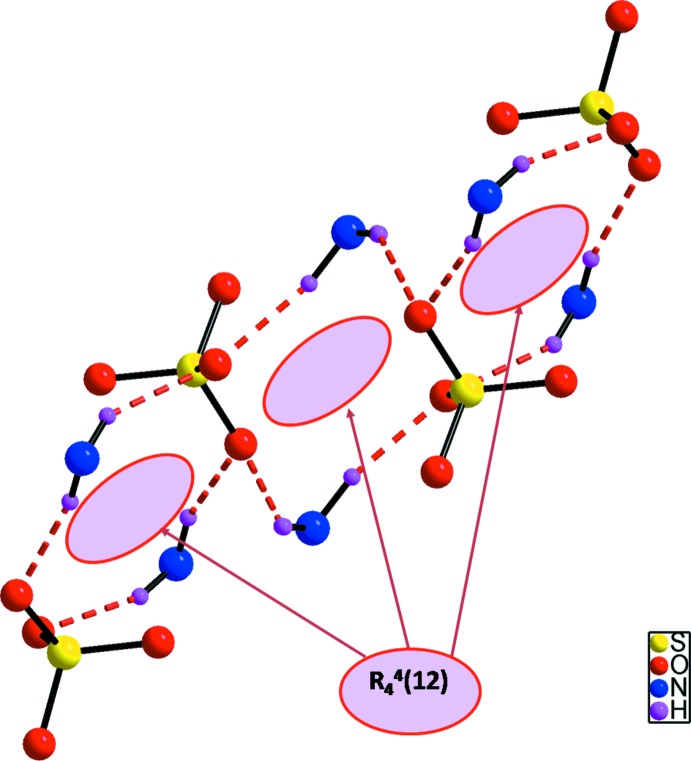
The 12-membered ring motif 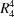
(12) in (I). C atoms have been omitted for clarity.

**Figure 3:**
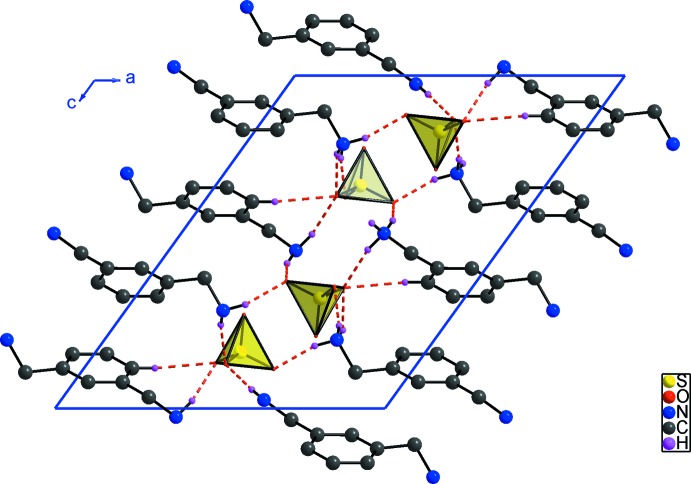
Projection of (I) along the *b* axis. H atoms not involved in hydrogen bonding have been omitted.

The packing for (I) generates rings with an 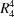
(12) motif (Fig. 2 ▸) and the overall structure of the title compound consists of infinite sheets of organic and inorganic entities propagating parallel to (100). Each organic dication is connected to six different sulfate anions *via* N—H⋯O and C—H⋯O hydrogen bonds, forming a three-dimensional supra­molecular network (Fig. 3 ▸).

The inter-planar distance between nearby benzene rings in the crystal structure is in the vicinity of 4.63 Å, which is much longer than 3.80 Å, value required for the formation of π–π inter­actions (Janiak, 2000 ▸).

## Hirshfeld analysis   

**Figure 4:**
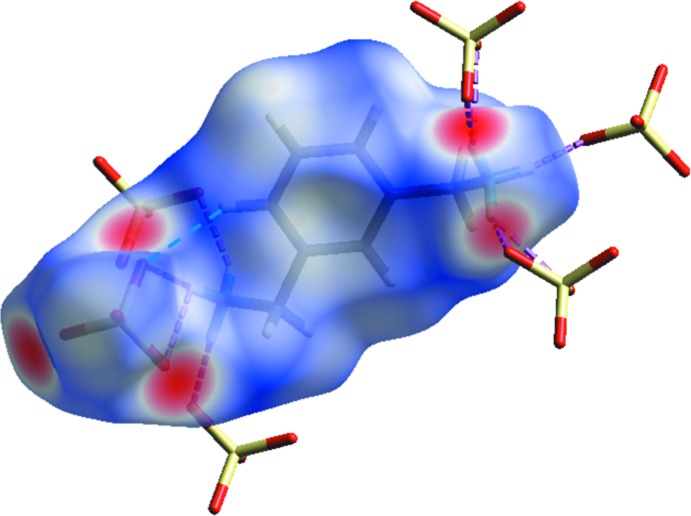
Hirshfeld surface mapped over *d*_norm_ showing hydrogen bonds with neighbouring sulfate groups. The surfaces are shown as transparent to allow visualization of the orientation and conformation of the functional groups. N—H⋯O and C—H⋯O hydrogen bonds are represented by red and blue dotted lines, respectively.

**Figure 5:**
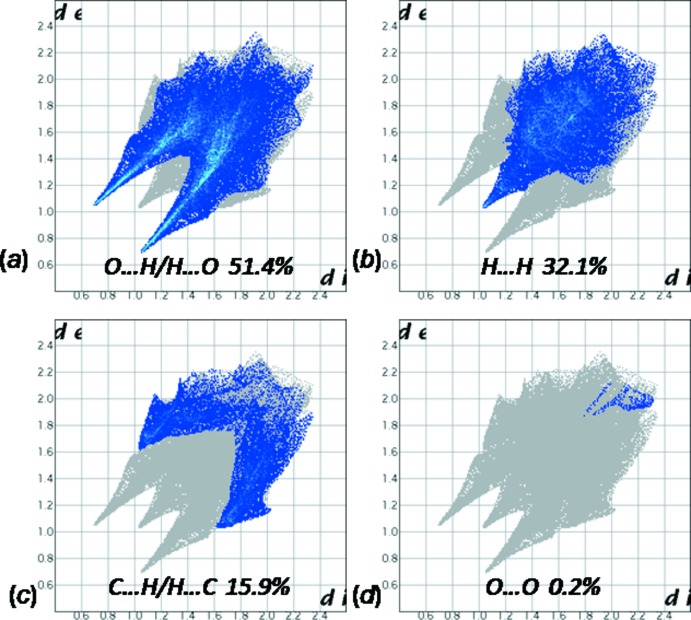
Fingerprint plots of the major contacts: (*a*) H⋯O, (*b*) H⋯H, (*c*) C⋯H and (*d*) O⋯O.

The three-dimensional Hirshfeld surfaces and two-dimensional fingerprint plots of (I) were prepared using *CrystalExplorer* (Wolff *et al.*, 2012 ▸) and are shown in Fig. 4 ▸ and Fig. 5 ▸, respectively.

The O⋯H/H⋯O contacts, which are attributed to N—H⋯O and C—H⋯O hydrogen-bonding inter­actions, appear as two sharp symmetric spikes in the two-dimensional fingerprint maps with a prominent long spike at *d*_e_ + *d*_i_ = 1.8 Å. They have the most significant contribution to the total Hirshfeld surfaces (51.4%). The H⋯H contacts appear in the middle of the scattered points in the two-dimensional fingerprint maps with a single broad peak at *d*_e_ = *d*_i_ = 1 Å and a percentage contribution of 32.1%. The 15.9% contribution from the C⋯H/H⋯C contacts to the Hirshfeld surface, generally slightly favoured in a sample of CH aromatic mol­ecules, results in a symmetric pair of wings, Fig. 5 ▸ *c*. The O⋯O contacts, which represent only 0.2% of the Hirshfeld surface, Fig. 5 ▸ *d*, are extremely impoverished in the crystal (enrichment ratio *E*_OO_ = 0.03) (Jelsch *et al.* 2014 ▸), as the oxygen atoms bound to sulfur and the SO_4_ group as a whole are electronegative, therefore the O⋯O contacts are electrostatically repulsive.

## Synthesis and crystallization   

Equimolar solutions of *m*-xylylenedi­amine dissolved in methanol and aqueous sulfuric acid were mixed together and stirred for about 1 h. Crystals of (I) were formed as the solvent evaporated over a few days at room temperature: these were filtered off, dried and repeatedly recrystallized as colourless prisms to enhance the purity of the product.

## Refinement   

Crystal data, data collection and structure refinement details are summarized in Table 2 ▸. H atoms bonded to N atoms were located from a difference map and were allowed to refine. The rest of the H atoms were treated as riding, with C—H = 0.93 Å (aromatic) or 0.97 Å (methyl­ene) with *U*_iso_(H) = 1.2*U*_eq_(C).

## Supplementary Material

Crystal structure: contains datablock(s) I. DOI: 10.1107/S2056989016006940/hb7579sup1.cif

Structure factors: contains datablock(s) I. DOI: 10.1107/S2056989016006940/hb7579Isup2.hkl

Supporting information file. DOI: 10.1107/S2056989016006940/hb7579Isup3.cml

CCDC reference: 1476189

Additional supporting information:  crystallographic information; 3D view; checkCIF report

## Figures and Tables

**Table 1:** Hydrogen-bond geometry (Å, °)

*D*—H⋯*A*	*D*—H	H⋯*A*	*D*⋯*A*	*D*—H⋯*A*
N1—H1*N*1⋯O4^i^	0.88 (2)	1.88 (2)	2.7271 (17)	160.1 (19)
N1—H1*N*1⋯O2^i^	0.88 (2)	2.54 (2)	3.1461 (18)	126.1 (16)
N1—H2*N*1⋯O1^ii^	0.90 (3)	1.85 (3)	2.7191 (17)	162 (2)
N1—H3*N*1⋯O3^iii^	0.88 (3)	2.03 (2)	2.8264 (17)	150 (2)
N1—H3*N*1⋯O2^iii^	0.88 (3)	2.54 (2)	3.1733 (18)	129.4 (18)
N2—H1*N*2⋯O4^iv^	0.84 (2)	1.97 (2)	2.8096 (17)	177 (2)
N2—H2*N*2⋯O1^v^	0.80 (3)	2.26 (3)	2.9537 (18)	145 (2)
N2—H3*N*2⋯O3	1.00 (3)	1.92 (3)	2.9021 (19)	168 (3)
N2—H3*N*2⋯O4	1.00 (3)	2.52 (3)	3.0502 (18)	113 (2)
C5—H5⋯O3	0.93	2.47	3.3050 (17)	150

Symmetry codes: (i) 
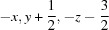
; (ii) 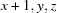; (iii) 
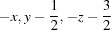
; (iv) 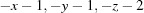; (v) 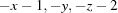.

**Table 2:** Experimental details

Crystal data	
Chemical formula	C_8_H_14_N_2_^2+^·SO_4_^2−^
*M*_r_	234.27
Crystal system, space group	Monoclinic, *P*2_1_/*c*
Temperature (K)	150
*a*, *b*, *c* (Å)	12.841 (1), 6.0989 (5), 15.9642 (9)
β (°)	125.791 (4)
*V* (Å^3^)	1014.15 (13)
*Z*	4
Radiation type	Mo *K*α
μ (mm^−1^)	0.32
Crystal size (mm)	0.56 × 0.44 × 0.30

Data collection	
Diffractometer	Bruker APEXII
Absorption correction	Multi-scan (*SADABS*; Bruker, 2014 ▸)
*T*_min_, *T*_max_	0.735, 0.910
No. of measured, independent and observed [*I* > 2σ(*I*)] reflections	10992, 2293, 2131
*R*_int_	0.048
(sin θ/λ)_max_ (Å^−1^)	0.649

Refinement	
*R*[*F* ^2^ > 2σ(*F* ^2^)], *wR*(*F* ^2^), *S*	0.038, 0.114, 1.14
No. of reflections	2293
No. of parameters	160
H-atom treatment	H atoms treated by a mixture of independent and constrained refinement
Δρ_max_, Δρ_min_ (e Å^−3^)	0.38, −0.61

Computer programs: *APEX2* (Bruker, 2014 ▸) and *SAINT* (Bruker, 2014 ▸), *XPREP* (Sheldrick, 2015 ▸), *SIR97* (Altomare *et al.*, 1999 ▸), *SHELXL2014/7* (Sheldrick, 2015 ▸) and *ORTEP-3 for Windows* and *WinGX* publication routines (Farrugia, 2012 ▸).

## supplementary crystallographic information

### Crystal data

**Table:**

C_8_H_14_N_2_^2+^·SO_4_^2^^−^	*F*(000) = 496
*M**_r_* = 234.27	*D*_x_ = 1.534 Mg m^−^^3^
Monoclinic, *P*2_1_/*c*	Mo *K*α radiation, λ = 0.71073 Å
*a* = 12.841 (1) Å	Cell parameters from 9927 reflections
*b* = 6.0989 (5) Å	θ = 3.7–27.5°
*c* = 15.9642 (9) Å	µ = 0.32 mm^−^^1^
β = 125.791 (4)°	*T* = 150 K
*V* = 1014.15 (13) Å^3^	Prism, colourless
*Z* = 4	0.56 × 0.44 × 0.30 mm

### Data collection

**Table:**

APEXII, Bruker-AXS diffractometer	2293 independent reflections
Radiation source: fine-focus sealed tube	2131 reflections with *I* > 2σ(*I*)
Graphite monochromator	*R*_int_ = 0.048
CCD rotation images, thin slices scans	θ_max_ = 27.5°, θ_min_ = 3.2°
Absorption correction: multi-scan (*SADABS*; Bruker, 2014)	*h* = −16→16
*T*_min_ = 0.735, *T*_max_ = 0.910	*k* = −7→7
10992 measured reflections	*l* = −17→17

### Refinement

**Table:**

Refinement on *F*^2^	Primary atom site location: structure-invariant direct methods
Least-squares matrix: full	Secondary atom site location: difference Fourier map
*R*[*F*^2^ > 2σ(*F*^2^)] = 0.038	Hydrogen site location: inferred from neighbouring sites
*wR*(*F*^2^) = 0.114	H atoms treated by a mixture of independent and constrained refinement
*S* = 1.14	*w* = 1/[σ^2^(*F*_o_^2^) + (0.0639*P*)^2^ + 0.4545*P*] where *P* = (*F*_o_^2^ + 2*F*_c_^2^)/3
2293 reflections	(Δ/σ)_max_ = 0.001
160 parameters	Δρ_max_ = 0.38 e Å^−^^3^
0 restraints	Δρ_min_ = −0.61 e Å^−^^3^

### Special details

**Table:**

Geometry. All esds (except the esd in the dihedral angle between two l.s. planes) are estimated using the full covariance matrix. The cell esds are taken into account individually in the estimation of esds in distances, angles and torsion angles; correlations between esds in cell parameters are only used when they are defined by crystal symmetry. An approximate (isotropic) treatment of cell esds is used for estimating esds involving l.s. planes.
Refinement. Refinement of F^2^ against ALL reflections. The weighted R-factor wR and goodness of fit S are based on F^2^, conventional R-factors R are based on F, with F set to zero for negative F^2^. The threshold expression of F^2^ > 2sigma(F^2^) is used only for calculating R-factors(gt) etc. and is not relevant to the choice of reflections for refinement. R-factors based on F^2^ are statistically about twice as large as those based on F, and R- factors based on ALL data will be even larger.

### Fractional atomic coordinates and isotropic or equivalent isotropic displacement parameters (Å^2^)

**Table:**

	*x*	*y*	*z*	*U*_iso_*/*U*_eq_	
S	−0.43829 (3)	−0.22666 (6)	−0.83072 (3)	0.01170 (15)	
O1	−0.57682 (10)	−0.1916 (2)	−0.88190 (9)	0.0190 (3)	
O2	−0.36751 (12)	−0.22945 (19)	−0.71797 (9)	0.0236 (3)	
O3	−0.38903 (10)	−0.04803 (18)	−0.86220 (9)	0.0187 (3)	
O4	−0.41947 (10)	−0.43848 (17)	−0.86666 (8)	0.0155 (2)	
N1	0.29551 (12)	−0.2271 (2)	−0.79401 (10)	0.0131 (3)	
C1	0.16854 (14)	−0.1870 (4)	−0.89148 (12)	0.0256 (4)	
H1A	0.1423	−0.3180	−0.9338	0.031*	
H1B	0.1768	−0.0699	−0.9284	0.031*	
C2	0.06392 (13)	−0.1263 (3)	−0.87945 (11)	0.0158 (3)	
C3	0.06536 (13)	0.0745 (3)	−0.83697 (10)	0.0161 (3)	
H3	0.1357	0.1674	−0.8089	0.019*	
C4	−0.03879 (13)	0.1353 (2)	−0.83668 (11)	0.0147 (3)	
H4	−0.0374	0.2689	−0.8080	0.018*	
C5	−0.14527 (12)	−0.0016 (2)	−0.87887 (10)	0.0125 (3)	
H5	−0.2147	0.0417	−0.8789	0.015*	
C6	−0.14779 (13)	−0.2033 (2)	−0.92106 (11)	0.0114 (3)	
C7	−0.04235 (14)	−0.2636 (2)	−0.92037 (12)	0.0150 (3)	
H7	−0.0430	−0.3985	−0.9478	0.018*	
C8	−0.25953 (13)	−0.3597 (2)	−0.96596 (12)	0.0160 (3)	
H8A	−0.2567	−0.4632	−1.0107	0.019*	
H8B	−0.2511	−0.4421	−0.9103	0.019*	
N2	−0.38513 (12)	−0.2467 (2)	−1.02553 (12)	0.0175 (3)	
H1N1	0.3194 (19)	−0.114 (4)	−0.7518 (16)	0.020 (5)*	
H2N1	0.350 (2)	−0.234 (3)	−0.8120 (18)	0.027 (6)*	
H3N1	0.299 (2)	−0.352 (4)	−0.7644 (17)	0.029 (6)*	
H1N2	−0.445 (2)	−0.340 (4)	−1.0567 (16)	0.021 (5)*	
H2N2	−0.387 (2)	−0.161 (5)	−1.064 (2)	0.039 (7)*	
H3N2	−0.399 (3)	−0.172 (5)	−0.977 (2)	0.059 (8)*	

### Atomic displacement parameters (Å^2^)

**Table:**

	*U*^11^	*U*^22^	*U*^33^	*U*^12^	*U*^13^	*U*^23^
S	0.0084 (2)	0.0123 (2)	0.0163 (2)	−0.00019 (11)	0.00831 (17)	−0.00144 (11)
O1	0.0099 (5)	0.0250 (6)	0.0266 (6)	0.0029 (4)	0.0133 (5)	0.0046 (5)
O2	0.0256 (6)	0.0229 (6)	0.0167 (6)	0.0027 (5)	0.0092 (5)	−0.0022 (4)
O3	0.0199 (5)	0.0146 (5)	0.0308 (6)	−0.0049 (4)	0.0201 (5)	−0.0037 (4)
O4	0.0148 (5)	0.0134 (5)	0.0212 (5)	−0.0007 (4)	0.0122 (4)	−0.0036 (4)
N1	0.0098 (6)	0.0150 (6)	0.0167 (6)	0.0016 (4)	0.0091 (5)	0.0017 (5)
C1	0.0064 (6)	0.0557 (12)	0.0145 (7)	−0.0006 (7)	0.0061 (6)	−0.0075 (7)
C2	0.0066 (6)	0.0296 (8)	0.0114 (6)	−0.0004 (5)	0.0054 (5)	−0.0016 (6)
C3	0.0081 (6)	0.0241 (8)	0.0137 (6)	−0.0050 (5)	0.0050 (5)	−0.0007 (5)
C4	0.0129 (6)	0.0159 (7)	0.0138 (6)	−0.0015 (5)	0.0070 (5)	−0.0016 (5)
C5	0.0092 (6)	0.0168 (7)	0.0130 (6)	0.0012 (5)	0.0074 (5)	0.0004 (5)
C6	0.0063 (6)	0.0161 (7)	0.0122 (6)	−0.0003 (5)	0.0056 (5)	0.0004 (5)
C7	0.0088 (6)	0.0209 (7)	0.0151 (7)	0.0006 (5)	0.0069 (6)	−0.0044 (5)
C8	0.0079 (6)	0.0138 (7)	0.0247 (7)	−0.0008 (5)	0.0087 (6)	−0.0035 (6)
N2	0.0066 (6)	0.0187 (7)	0.0211 (7)	−0.0022 (5)	0.0047 (5)	0.0051 (5)

### Geometric parameters (Å, º)

**Table:**

S—O2	1.4673 (12)	C3—H3	0.9300
S—O1	1.4756 (10)	C4—C5	1.3941 (19)
S—O3	1.4871 (11)	C4—H4	0.9300
S—O4	1.4895 (11)	C5—C6	1.393 (2)
N1—C1	1.4738 (19)	C5—H5	0.9300
N1—H1N1	0.88 (2)	C6—C7	1.3971 (19)
N1—H2N1	0.90 (3)	C6—C8	1.5100 (19)
N1—H3N1	0.88 (3)	C7—H7	0.9300
C1—C2	1.510 (2)	C8—N2	1.4787 (18)
C1—H1A	0.9700	C8—H8A	0.9700
C1—H1B	0.9700	C8—H8B	0.9700
C2—C3	1.395 (2)	N2—H1N2	0.84 (2)
C2—C7	1.395 (2)	N2—H2N2	0.80 (3)
C3—C4	1.391 (2)	N2—H3N2	1.00 (3)
			
O2—S—O1	111.24 (7)	C3—C4—C5	120.81 (14)
O2—S—O3	110.02 (7)	C3—C4—H4	119.6
O1—S—O3	108.67 (7)	C5—C4—H4	119.6
O2—S—O4	109.80 (6)	C6—C5—C4	120.08 (13)
O1—S—O4	109.08 (7)	C6—C5—H5	120.0
O3—S—O4	107.96 (6)	C4—C5—H5	120.0
C1—N1—H1N1	110.6 (13)	C5—C6—C7	118.71 (13)
C1—N1—H2N1	104.9 (15)	C5—C6—C8	122.29 (12)
H1N1—N1—H2N1	107.1 (19)	C7—C6—C8	119.00 (13)
C1—N1—H3N1	112.8 (15)	C2—C7—C6	121.56 (14)
H1N1—N1—H3N1	112 (2)	C2—C7—H7	119.2
H2N1—N1—H3N1	109 (2)	C6—C7—H7	119.2
N1—C1—C2	115.06 (13)	N2—C8—C6	112.76 (12)
N1—C1—H1A	108.5	N2—C8—H8A	109.0
C2—C1—H1A	108.5	C6—C8—H8A	109.0
N1—C1—H1B	108.5	N2—C8—H8B	109.0
C2—C1—H1B	108.5	C6—C8—H8B	109.0
H1A—C1—H1B	107.5	H8A—C8—H8B	107.8
C3—C2—C7	119.07 (13)	C8—N2—H1N2	109.8 (15)
C3—C2—C1	121.14 (14)	C8—N2—H2N2	108.8 (18)
C7—C2—C1	119.50 (15)	H1N2—N2—H2N2	112 (2)
C4—C3—C2	119.76 (13)	C8—N2—H3N2	109.3 (18)
C4—C3—H3	120.1	H1N2—N2—H3N2	105 (2)
C2—C3—H3	120.1	H2N2—N2—H3N2	112 (3)

### Hydrogen-bond geometry (Å, º)

**Table:**

*D*—H···*A*	*D*—H	H···*A*	*D*···*A*	*D*—H···*A*
N1—H1*N*1···O4^i^	0.88 (2)	1.88 (2)	2.7271 (17)	160.1 (19)
N1—H1*N*1···O2^i^	0.88 (2)	2.54 (2)	3.1461 (18)	126.1 (16)
N1—H2*N*1···O1^ii^	0.90 (3)	1.85 (3)	2.7191 (17)	162 (2)
N1—H3*N*1···O3^iii^	0.88 (3)	2.03 (2)	2.8264 (17)	150 (2)
N1—H3*N*1···O2^iii^	0.88 (3)	2.54 (2)	3.1733 (18)	129.4 (18)
N2—H1*N*2···O4^iv^	0.84 (2)	1.97 (2)	2.8096 (17)	177 (2)
N2—H2*N*2···O1^v^	0.80 (3)	2.26 (3)	2.9537 (18)	145 (2)
N2—H3*N*2···O3	1.00 (3)	1.92 (3)	2.9021 (19)	168 (3)
N2—H3*N*2···O4	1.00 (3)	2.52 (3)	3.0502 (18)	113 (2)
C5—H5···O3	0.93	2.47	3.3050 (17)	150

Symmetry codes: (i) −*x*, *y*+1/2, −*z*−3/2; (ii) *x*+1, *y*, *z*; (iii) −*x*, *y*−1/2, −*z*−3/2; (iv) −*x*−1, −*y*−1, −*z*−2; (v) −*x*−1, −*y*, −*z*−2.
